# Food Security—A Commentary: What Is It and Why Is It So Complicated?

**DOI:** 10.3390/foods1010018

**Published:** 2012-12-03

**Authors:** Mark Gibson

**Affiliations:** Institute for Tourism Studies (IFT), Colina de Mong-Ha, Macao, China; E-Mail: markgibson@ift.edu.mo; Tel.: +853-85061-459; Fax: +853-85983-035

**Keywords:** food security, famine, hunger, malnutrition, under-nutrition, poverty, sustainability, food production

## Abstract

Every year over 10 million people die of hunger and hunger related diseases. Nearly six million of these are children under the age of five; that is one child’s death approximately every six seconds. Understanding how this still occurs amid the ever increasing social enlightenment of the 21st century—and under the auspices of a vigilant global developmental community—is one of the key challenges of our time. The science of food security aims to address such concerns. By understanding the multiplicity of the phenomenon, practitioners of global multilateral hegemony seek to shape appropriate policy to address these issues. The difficulty however is that the phenomenon is increasingly wrapped up inside an ever growing bundle of societal aspirations including *inter-alia* under-nutrition, poverty, sustainability, free trade, national self sufficiency, reducing female subjugation and so on. Any solutions therefore, involve fully understanding just what is indeed included, implied, understood or excluded within the food security catchall. Indeed, until such time as consensus can be found that adequately binds the phenomenon within a fixed delineated concept, current efforts to address the multitude of often divergent threads only serves to dilute efforts and confound attempts to once-and-for-all bring these unacceptable figures under control.

## 1. Introduction

Over the last 40 years (since adequate records have been kept), the numbers of hungry and malnourished people around the world have hovered between 800 million and 1.2 billion people [1]. Indeed, these numbers are staggering and one does not need to be a humanitarian to appreciate that that such figures are unacceptably high by anybody’s standards. Food security then, briefly and for introductory purposes, can be thought of as understanding how and why this phenomenon exists and continues to exist to the extent that it does. From this it follows that the notion of food security is a global phenomenon impinging on every human being’s daily life.

Yet despite the global reach of the phenomenon and the deceptively simple introductory definition, food security still engenders widespread misconception and misunderstanding [2]. Even with much talk in the media of late, it would seem that the term is still commonly being confused with terms like “food safety” for instance; these are two very separate issues [3]. Another frequently perpetuated misconception is that food insecurity is the sole preserve of developing countries [2]. Once again however, the reality is far different, on the contrary in fact; it can be seen that many developed economies too suffer the same inequalities of food and by extension—nutritional distribution as the more developing regions; although granted, largely to a lesser extent [2].

## 2. Food Security: What Is Food Security?

So, in an attempt to add to the literature that seeks to set the record straight, we once again ask—what is food security? In answer, at its very basic it means *regularly* having enough food to eat; not just for today or tomorrow, but also next month and next year. All very straightforward—so why the ongoing confusion? To help answer this, consider the various questions this apparently simple concept raises. As a starting point, for simplification; and without getting too far drawn into the nuances and complexities of the subject, the Food and Agriculture Organization (FAO) of the United Nations suggests, that food security is the product of food availability, food access, stability of supplies and biological utilisation [4].

### 2.1. Availability

Considering the dimension of availability; food is provided through one of two means—domestic production and/or imports. This requires thought and consideration to be given to the physical availability of food at farms and in local markets. In turn this is predicated on well-functioning market infrastructures with adequate road and rail networks, as well as ensuring adequate storage and processing technologies [5,6].

### 2.2. Access

Food access entails ensuring people have adequate access, both physical and economic to food through growing it; purchasing it; being gifted it; bartering or trading for it *etc. *[5]*.* This concept can be thought of as a package of entitlements that allows an individual to acquire and maintain appropriate foods for an adequate diet and nutritional level. This might be directly obtained as mentioned through own-grown produce, earning sufficient income, barter and exchange; or indirectly via social arrangements either at the community or national levels such as through family, welfare systems, traditional rights, access to common resources and of course emergency food aid [7,8]. 

### 2.3. Stability

When talking of stability, although not a new idea, the realisation that food security can be lost as well as gained is of increasing concern within the food security debate [9]. As a result, the notion of risk management is gaining much credibility as a tool in the fight against hunger. Such consideration involves issues of stability and vulnerability; this can be of the wider economy in general; of livelihoods in particular; of incomes, or even of food supplies themselves concentrating on shocks, sudden or otherwise such as floods, droughts or pests *etc.* [9].

### 2.4. Biological Utilisation

The last concept is the notion of biological utilisation and despite the “novelty” of the nomenclature, the idea itself is not new; utilisation is simply the ability of a person to optimally or at least effectively, absorb the food they eat. In turn research has shown that this ability is closely related to a person’s health status which, in turn is also predicated on important non-food inputs. Indeed it has been shown that optimum biological utilisation necessitates the need for proper health and child care; clean water and sanitation services; adequate knowledge of nutritional and physiological needs as well as the proper application of such knowledge [10,11].

## 3. Pandora’s Box

The above, although slightly more detailed still only scratches the surface of the concept of food security. Yet even in this incarnation, hints as to the breadth and the true extent of the multi-dimensionality of food security issues start to emerge. With this in mind, consider then the various differing definitions used to describe this phenomenon. First off, it is worth noting that even back in 1992 a thorough study by Maxwell and Frankenberger had already identified close to 200 separate definitions [12]. That aside, and sticking with the more widely accepted current institutional definitions, there are perhaps two major bodies whose definitions are commonly quoted; those of the United Nations (UN) and various bodies of the United States (US).

Firstly the UN’s definition which itself has had many incarnations over the years; and which incidentally is still being widely debated. Indeed as late as October 2012 the Committee on World Food Security (CFS) had attempted to, once again revise the terminology of their current definition to reflect popular progressive thinking [13]:

“Food and nutrition security exists when all people at all times have physical, social and economic access to food, which is safe and consumed in sufficient quantity and quality to meet their dietary needs and food preferences, and is supported by an environment of adequate sanitation, health services and care, allowing for a healthy and active life.”

Unfortunately however, these amendments were not endorsed due to being blocked by some countries. Alas despite their best efforts, the UN’s current definition remains that of the 2001 State of Food Insecurity report in which it suggested [14]:

“Food security (is) a situation that exists when all people, at all times, have physical, social and economic access to sufficient, safe and nutritious food that meets their dietary needs and food preferences for an active and healthy life.”

The US on the other hand employs several definitions depending on need and the many disparate institutional bodies. In general though, the US Department of Agriculture (USDA) normally focuses on national hunger issues while the US Agency for International Development (USAID) mainly operates with an international remit.

Firstly the USDA defines food security as [15,16]:

“Access by all people at all times to enough food for an active, healthy life. Food security includes at a minimum: (1) the ready availability of nutritionally adequate and safe foods, and (2) an assured ability to acquire acceptable foods in socially acceptable ways (e.g., without resorting to emergency food supplies, scavenging, stealing, or other coping strategies).”

The USAID on the other hand has several definitions depending on the particular purpose. USAID’s current general classification though is based on USAID Policy Determination #19 from 1992 which states [17]:

“When all people at all times have both physical and economic access to sufficient food to meet their dietary needs in order to lead a healthy and productive life.”

The US also have the Agricultural Trade Development and Assistance Act, or more commonly the Public Law 480 (PL 480) program which offers a more flexible definition to allow for a range of possible interventions. Again based on Policy Determination #19 food security is determined as [18]:

“Access by all people at all times to sufficient food and nutrition for a healthy and productive life.”

Furthermore the USDA Economic Research Service (ERS) also define the food insecure as those consuming less than 2100 kcal per day [19].

There are of course others including those of the European Union and Oxfam *etc.*, but the point is well made. Looking at these definitions perhaps the first thing to note is that they collectively represent the more convergent of the many definitions on offer. Yet even despite the certain striking similarities in overall conceptual design, in detail there are also many key differences. This is where food security begins to take on a complexity all of its own. For instance, both FAO and US PL480 include “all people at all times” and “safe” foods—no ambiguity there, but what of “sufficient, nutritious (FAO and USAID)” and “…nutritionally adequate (USDA)” foods [14,18]? What are the nutritional needs of humans? Is it a one-size-fits-all deal or are there different nutritional needs for different people? Even when this can be agreed upon, who then determines, or sets the standards? For that matter too, what is the difference between sufficient and adequate or is there a difference?

And what of the FAO’s “physical, social and economic access” dimensions [14]? Furthermore does the availability of food in sufficient quantity also include consideration of quality; if so, again to what or whose standards? Also what of the market mechanism itself? Is this to be governed by free trade or perhaps through government manipulation of such variables as agricultural subsidies, quota’s and import tariffs *etc.*; similarly, who sets food safety standards policies? Are there sufficient market infrastructures; are they well maintained? 

Moreover, while the FAO talk of “food preferences” [14] the USDA focuses on “acceptable foods” [15,16]—in this case is acceptable food, acceptable to the person (as in preference) or to the nutritional needs of the person (as in requirements)? Further while, the FAO and USDA talk of an “active and healthy life” [14,15,16], USAID and US PL 480 consider a “healthy and productive life” [18]; what is and how is an active, healthy or productive life determined, targeted for and measured? Lastly, one does not need to be an expert to understand that the USDA ERS’s blanket figure of less than 2100 kcal per day to represent the food insecure is somewhat potentially problematical. For some of course, such differences might merely be a matter of semantics but for others—policy makers, statisticians and the like—especially if policy is to be predicated on such concepts, such nuances need to be clarified to the nth degree.

### 3.1. It Just Gets Bigger

**Figure 1:**
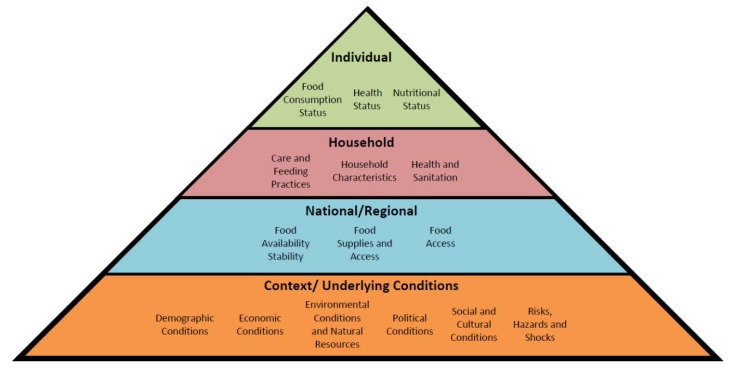
Different levels of food security. Based on ideas from the Committee on Food Security [13] and the FIVIMS initiative [20].

The questions are in fact ad-infinitum and yet this does not even begin to take into consideration the fact that such analysis is generated from a single viewpoint (that of the individual) and within a single dimension. In reality, if we were to factor in issues beyond this simple view we are then drawn into the realms of household and national or regional food insecurity as well as the different dimensions of temporary (temporal), chronic (continuous) or cyclical (seasonal) food insecurity. Indeed, one need only look at the conceptual ideas of the Inter-Agency Working Group (IAWG) on the Food Insecurity and Vulnerability Information and Mapping Systems (FIVMS), to garner a glimpse of just how exponentially complicated the subject can become. FIVIMS for instance, identify 15 “information domains” to aid in understanding the causes of poor food consumption and nutritional status [20]. These can be seen in Figure 1 and range from the individual to the household to the national; in turn these concepts are further underpinned by several contextual factors whose understanding and interplay are the subject of much research [20].

### 3.2. Different Things to Different People

Further compounding a full understanding of the subject is the fact that the phenomenon is often studied from a particular specialist perspective. Agriculturalists for instance might concentrate on increasing production through improved land management or through maximising crop yield potentials; prevention of disease; and pest control measures *etc.* This has wide implications for the agricultural community at large, for as expanding populations need to grow, so this may well require biotechnologies and other scientific disciplines to work closely with the agrarian community. In doing so further ethical questions are then brought to the debate regarding natural *versus* genetically modified organisms; and in such circumstances what of public perceptions and acceptance? 

Another interested group is the sociologists whose focus on food security issues might be through the lens of cultural and societal influences of insecurity; population growth and how poverty affects malnourishment; the psychology of income growth and changing dietary habits; or perhaps the dynamics of rural-urban change among others. Also within the sociological mix, considerations are made concerning the non-food aspects of good nutrition which may consist of among others, shelter, sanitation and education. And what of health? Links have been made between poor health, malnutrition and disease. This puts primary health care into the mix along with everything else. Moreover, from the sociological perspective, what of those that cannot provide for themselves; do we once again turn to the humanitarian sector or perhaps offer myriad social safety nets?

Politicians and economists too are not to be left out. This group might attempt to affect food security outcomes through policy driven by domestic or international necessity, political ambition or even public sentiment. Although, in this regard the big caveat here are the various economic and social trade-offs between policies which aim to balance needs with objectives that reflect the prevailing economic and political sentiments of the day.

## 4. Governance

Yet even amongst all these drivers we need to ask ourselves who is responsible for humanities security of food, is it the individual; the government or state; a multilateral body or institution? Are we to work collaboratively as nations—a global collective, or are the intrinsic self interests of nation states to be put before those of the wider community? In the wider debate do we provide solutions, or simply the means to solutions?

On this, many in fact see the United Nations (UN) as assuming the lead role in global governance along with the USA, various EU agencies and other small but influential think tanks, policy analysts and charitable organisations. Yet without global consensus on a single source of stewardship it becomes difficult to navigate the phenomenon and stay abreast of the latest research or understanding. Publications, journal articles, studies and consultations all take time to filter into general discussion. Then there are the conferences and symposia which debate everything from definitions to methodologies, from concepts to targets. Multilateral and unilateral agreements are made, decisions are questioned and policy strategised and all the while the latest ideological “breakthroughs” tabled for general acceptance might include or exclude previously contended issues. In this melee it becomes increasingly difficult to grasp the extant level of comprehension of the subject or to fully understand by what standards we are measuring, analysing or comparing data, ideas or current thinking. 

## 5. Conclusion

Understanding and promoting food security then, is as much about coming to grips with its nemesis—*food insecurity*. Through reverse engineering the social construct, students of the phenomenon aim to break down the problem into its component parts. Food insecurity itself is a multifaceted phenomenon involving many variables and existing on several typological planes which can and does originate from a plethora of possible causes. Moreover, understanding the notion draws on just as many disparate disciplines—from science and biotechnology to political ideology; and from social and economic developmental philosophies to environmentalism and more [5]. For some, food security represents the ability to trade, supply or simply purchase food in a global marketplace unimpeded by barriers. For others food security is seen as the right of a country to own its food sovereignty—its ability to directly or indirectly exercise control over its own food needs. Yet others still see hunger and nutrition issues as central to an individual’s basic human rights. 

On top of this, not nearly enough is known about how all these variables combine or interact to promote or deny food security. Some, like climate change might have obvious implications on the pattern of food production; or so we might think. But what of the other variables; how does the philosophy of economic development affect poverty alleviation and do we fully understand it? What of migration or urbanisation? How about natural resources or biofuels and what else might we not have considered?

From this it can be seen then that the seemingly simple notion of food security is very quickly complicated by difficult and uncomfortable realities; the salient point being that food insecurity is a multi-headed beast with many different masters [21,22]. Today it seems that, in and of itself, food security is being seen less and less in isolation of a much wider societal context. On this point and in a rare introspective, the FAO suggested that people have tended to apply contrasting diopolistic significance to the food security phenomenon, suggesting on the one hand, that it has been used as [23]:

“…little more than a proxy for chronic poverty…”

and on the other—a tendency to apply [23]:

“…an all-encompassing definition, which ensures that the concept is morally unimpeachable and politically acceptable, but unrealistically broad.”

Indeed reflecting on this point, there appears to be increasing convergence of ideology whereby security of food is being viewed more and more as a constituent part of the broader concept of the social welfare construct including *inter-alia*, nutrition security [1], health care, poverty alleviation, education and human rights [24,25]. 

Yet while laudable goals in and of themselves, collective progress continues to be wrapped up in the difficulties of conceptual and practical interpretation. Indeed, with such widely divergent remits, security of food continues to suffer from misunderstanding and misconception resulting in ongoing misdirection and dilution of effort and by extension—results. Indeed, until such time as the food security concept stops being all things to all people; or until such time as the international community can properly and adequately re-focus the concept into a single consensual goal, food security is likely to continue to suffer the unacceptably high recurring figures of malnutrition.
